# Exploring the urban gradient in population health: insights from satellite-derived urbanicity classes across multiple countries and years in sub-Saharan Africa

**DOI:** 10.1136/bmjgh-2023-013471

**Published:** 2023-10-21

**Authors:** Peter M Macharia, Jessie Pinchoff, Cameron Taylor, Lenka Beňová

**Affiliations:** 1 Department of Public Health, Institute of Tropical Medicine, Antwerpen, Belgium; 2 Population and Health Impact Surveillance Group, KEMRI-Wellcome Trust Research Programme, Nairobi, Kenya; 3 Social and Behavioral Sciences Research, Population Council, New York, New York, USA; 4 The DHS Program, ICF, Rockville, Maryland, USA; 5 Medicine and Health Sciences, University of Antwerp, Antwerpen, Belgium; 6 Faculty of Epidemiology and Population Health, London School of Hygiene & Tropical Medicine, London, UK

**Keywords:** geographic information systems, indices of health and disease and standardisation of rates, maternal health, public health, epidemiology

## Abstract

The demographic, ecological and socioeconomic changes associated with urbanisation are linked to changes in disease incidence, health service provision and mortality. These effects are heterogeneous between and within urban areas, yet without a clear definition of what constitutes an ‘urban’ area, their measurement and comparison are constrained. The definitions used vary between countries and over time hindering analyses of the relationship between urbanisation and health outcomes, evaluation of policy actions and results in uncertainties in estimated differences. While a binary urban-rural designation fails to capture the complexities of the urban-rural continuum, satellite data augmented with models of population density and built-up areas offer an opportunity to develop an objective, comparable and continuous measure which captures urbanisation gradient at high spatial resolution. We examine the urban gradient within the context of population health. We compare the categorisation of urban and rural areas (defined by national statistical offices) used in household surveys in sub-Saharan Africa (SSA) to an urban-rural gradient derived from augmented satellite data within a geospatial framework. Using nine Demographic and Health Surveys (DHS) conducted between 2005 and 2019 in six SSA countries, we then assess the extent of misalignment between urbanicity based on DHS categorisation compared with a satellite-derived measure, while discussing the implications on the coverage of key maternal health indicators. The proposed indicator provides a useful supplement to country-specific urbanicity definitions and reveals new health dynamics along the rural-urban gradient. Satellite-derived urbanicity measures will need frequent updates to align with years when household surveys are conducted.

**Summary box**

- Urbanisation is a complex socioeconomic process of expansion/creation of densely populated human settlements and is associated with various health outcomes.
- National statistical offices’ definitions of areas as urban or rural which is used in household surveys differ between countries and within countries over time.
- A binary urban-rural definition fails to account for the urban-rural continuum and the complex relationships between urbanicity and health outcomes.
- Satellite-derived data augmented with data on population density and built-up areas can provide comparable, accurate, timely and continuous measures of the urban gradient at a high spatial resolution, including variations *within* urban areas.
- Researchers and stakeholders working on population health in urban settings should complement national statistical offices’ definitions of urban areas by incorporating alternative data, such as satellite imagery combined with other auxiliary datasets, to facilitate a robust analysis of urbanisation and health outcomes.

## Introduction

Urbanisation is a complex socioeconomic process which involves people moving to urban areas, faster population growth in urban centres and recategorisation of areas to *urban* as their populations grow.^1–3^ Two-thirds of the world’s population will live in urban settings by 2050 and the fastest rates of urbanisation are in Africa and Asia where nearly 90% of these additional 2.5 billion urban residents will be concentrated.^1^ Urbanisation is fastest in countries where health indicators are worst. Urbanisation affects the spatial distribution and characteristics of the population in both urban and rural areas, including their occupations, lifestyles, culture and behaviour.^1 2^ The demographic, ecological and socioeconomic transformations that come with urbanisation are associated with various health outcomes and interact with the ongoing epidemiological transition.

Urban residence is associated with mortality rates for adults, children under 5 and newborns,^4–7^ all of which are used as proxy measures of population health. Additionally, urbanisation influences other health outcomes, such as disease burden, immunisation rates and the provision and use of reproductive and maternal healthcare, including childbirth care and associated quality.^8–10^ Urbanisation impacts health outcomes directly and through other socioeconomic pathways such as education, empowerment, environment and the economy. Therefore, how we tackle health issues to achieve Sustainable Development Goals (SDGs) by 2030 must deeply consider the realities of rapid urbanisation, including poverty, housing and women’s status/rights.

Robust datasets depicting urban areas play a crucial role in enhancing our understanding of urbanisation, or urbanicity—the nature of urban environments—and its relationship with health outcomes.^11^ Therefore, how urbanicity is measured will affect research findings and subsequent actions. In sub-Saharan Africa (SSA), disease registries for population-based health data are either incomplete or non-existent. Consequently, population health research and policy-making heavily rely on nationally representative household surveys, especially Demographic and Health Surveys (DHS)^12^ and Multiple Indicator Cluster Surveys (MICS).^13^ These surveys gather data at the enumeration area (EA) level which is labelled either as urban or rural. An EA is a counting unit defined during a census and may refer to a city block, apartment building, village or group of villages.^12^ EAs represent urbanicity through an urban-rural dichotomy determined by the respective countries and adopted by the DHS or MICS Program.^14^ Critically, there is no universally accepted definition of ‘urban’ across countries. For example, most countries use a population threshold, yet the size of defined urban areas can vary from 200 in Denmark to 100 000 people in China^15^ (online supplemental data). Consequently, definitions vary between countries, and even within a single country the definition is revised over time.^16^

10.1136/bmjgh-2023-013471.supp1 Supplementary data

Non-standardised definitions of urban areas lead to inconsistency in the classification of EAs as either urban or rural in the DHS.^14^ This hinders comparative analyses of the relationship between urbanicity and health outcomes, and evaluation of policies between countries.^11 17 18^ These compromise benchmarking progress and the ability to meaningfully compare SDG indicators for urban areas.^15^ For instance, research findings indicating higher mortality rates in urban areas might be an artefact of how an urban area is defined.^6^ Current and historical facts concerning health indicators that are intertwined with urbanisation patterns across different countries may be biased due to measurement errors when labelling an area as urban.^19^

Beyond the lack of consistent definitions, the oversimplified urban-rural dichotomy in household surveys and other applications are widely recognised as inadequate.^14 16 20 21^ They obscure the complex and often non-linear relationships between various degrees of urbanisation and health outcomes,^20–22^ which in turn conceal population health inequities. The inequities arise from the variation of demographic, ecological and socioeconomic factors such as unregulated built environments, congestion and informal settlements^16 18 23 24^ within urban areas. This means the intricate dynamics that exist between degrees of urbanicity (a densely populated inner city is not the same as a semiurban suburb) and various health indicators are overlooked. Ideally, the continuum should be dynamic, capturing temporal changes and representing a spectrum of gradations from rural to urban environments that are applicable in the context of population health.^20 25^

Urban areas in SSA have historically been considered to have better health outcomes than rural areas due to better infrastructure, easier access to healthcare and improved educational attainment in urban areas compared with rural ones.^7^ However, current evidence shows that in SSA, the urban *advantage* might be diminishing or reversing in some cases.^4 6 8^ Urban areas are becoming ‘the new rural areas’ in terms of disadvantages as they are increasingly characterised by poor planning, informal settlements and traffic congestion.^23^ Thus, understanding the urban gradient is important as evidently, all is not equal within urban areas. Burdens are expected to be concentrated in a few *hot spot* areas; identification of these areas and directing suitable interventions ensures equitable resource allocation and understanding of why there is a reversal. Further, understanding the reversals or in fact determining to what extent these are artefacts of how urban areas are defined,^5^ and if true, to identify potential causal factors which can be effectively addressed by policies and interventions will require an objective defined urban gradient.

Therefore, appeals have been made for further research enhancing the understanding and measurement of urbanicity gradients^11 20 22 26 27^ to better capture within-urban variation and inequalities. To advance the creation of a framework for classifying EAs based on urbanicity, the DHS Program and researchers proposed to contrast urbanicity-related factors with the conventional urban-rural classification.^14 28^ In this analysis article, we interrogate the urban-rural classification as used by the DHS and summarise alternative methods to the conventional urban-rural dichotomy. Through the application of one of these alternatives (satellite data), we create an urban-rural gradient at EA level across nine DHS surveys conducted in six countries. We compare this gradient with DHS urban-rural classifications and assess how the alternative classification modifies the findings on coverage of key maternal health indicators. We selected six countries in SSA, namely Cameroon (2004 and 2011), Ethiopia (2016 and 2019), Ghana (2014), Guinea (2005), Kenya (2008/2009 and 2014) and Zambia (2013/2014), based on regional representation, availability of DHS data and the inclusion of countries with medium to large urban areas. The definitions of urban areas in each of these countries is outlined in the online supplemental data. For each country, we considered the most recent two DHS surveys provided its ±1 year of urbanisation data. Where the survey year and the DHS year did not match (±1 year) for the two most recent surveys, the third most recent DHS was considered. The data were available from the DHS website.

We make a case for a more sensitive, accurate, up-to-date, objective and continuous gradient of urbanicity to better understand and address health issues, given the diversity of urban settings. We expect this dialogue to prompt the research community to make informed choices when using the DHS urban-rural categories or determining the urbanisation continuum for population health.

## DHS and urbanicity

The DHS are nationally representative cross-sectional household surveys in which standard model questionnaires are adapted to each country’s context. These surveys, initiated in 1984, gather data on population health across more than 90 low and middle-income countries.^12^ The survey sampling design has two stages. The first stage involves selection of EAs while the second stage involves selection of households within the sampled EA. Each EA typically contains 20–30 households randomly selected to be surveyed from about 100–300 households per EA. DHS are also stratified by urban and rural locations and by geographical region and representative at these two domains.^12^

DHS collects coordinates for the sampled EAs, with a positional accuracy of 15 m.^29^ To protect and maintain the confidentiality of the respondents, the coordinates of the EAs are randomly displaced by 5 km for rural EAs (with 1% of the rural EAs displaced up to 10 km) and 2 km for urban EAs.^29^ The displaced (geoscrambled) coordinates have been available to the public since 2003. The displacement of cluster coordinates inevitably impacts spatial analyses, such as spatial prediction, proximity metrics and the derivation of different urbanicity classes,^29^ the subject of our analysis.

The definition of urban and rural used by the DHS is based on the designation from the national statistical office of the country in which a survey is being undertaken. These definitions are based either on objective criteria (such as administrative, population, functional or economic attributes), subjective criteria (such as centricity of a settlement or its infrastructure) or a mix of characteristics.^3 14 16 18 20 21 24 30 31^ Globally, the national definitions of ‘urban’ can be categorised into 10 broad groups based on 2005 data from the United Nations based on the subjective and objective criteria such as population size, economic activities, administrative facilities, structures and services.^16^ Specifically in Africa, the most widely used criteria are political/administrative (25 countries), population threshold (13 countries) and a hybrid (15 countries).^14 30^ Indeed, the distinction between urban and rural is somewhat arbitrary and influenced by cultural and political factors, hence the variation across countries.^18^

Studies of population health using datasets from these surveys are therefore limited to this simple urban-rural dichotomy which fails to acknowledge the urban-rural gradient that exists, ranging from remote rural areas and semiurban suburbs to core urban areas. While the boundaries as defined by governments based on various administrative, political and functional criteria appear precise and fixed,^14 30^ they do not accurately demarcate the physical space where interactions between economic and social agents occur (the lived environment).^19 32^ In addition, urban areas continually evolve, develop and grow beyond these political boundaries.^14 19 30^ Therefore, consistency within and across countries in defining urban areas is critical for population health.

## Alternative satellite-derived data products and models

Researchers have also made efforts (box 1) to establish definitions for urban areas both globally and comparatively. These alternative approaches use satellite-derived datasets, built environment, land use/cover classes and population density either independently or in conjunction.

Box 1 **Examples of alternative sources and measures of the urban gradient**

 **Degree of urbanisation (DoU)**: A global definition of *cities* (at least 50 000 inhabitants in contiguous dense grid cells), *towns* and *semidense areas* (at least 5000 inhabitants in contiguous grid cells with a density of ≥300 inhabitants per km^2^) and *rural areas* (low-density grid cells of <300 inhabitants per km^2^).^15 33^

**Functional urban area**: An extension of DoU, a database of metropolitan areas in the entire world comprising high-density urban centres with at least 50 000 people plus their surrounding commuting zones.^33 38 39^

**Figure 3:**
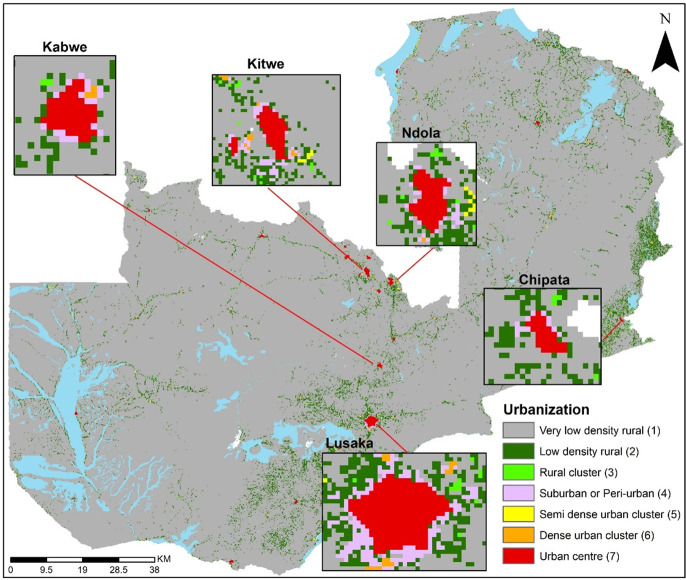
GHS-Settlement Model layer (GHS-SMOD) gridded urbanisation surface in Zambia, 2015.

**Global Human Settlement Layer (GHSL)**: The GHS-Settlement Model layer (GHS-SMOD) gridded surface is based on the distribution of population, built-up surfaces and the land surface based on satellite image and Open Street Map (OSM) data. The combination of these three spatial data layers based on the DoU concept^33 34^ results in GHS-SMOD at 1 km spatial resolution,^40^ as shown in figure 3 for Zambia. There are two levels of classification in the GHS-SMOD dataset. Level 1 encapsulates three classes (urban centre, urban cluster and rural grid cell) which are further broken down (level 2) into eight classes: urban centre, dense urban cluster, semidense urban cluster, sub-urban or periurban, rural cluster, low-density rural, very-low-density rural and water grid cells.

**Global Rural-Urban Mapping Project (GRUMP)**: Urban areas are defined based on night-time lights (NTL), buffered settlement centroids (where NTLs are not sufficiently bright) and human settlement points of cities and towns with populations of greater than 1000 persons.^41^ Liu and colleagues also provide an overview of intermediate-resolution global urban extent data products generated from satellite imagery including global urban built-up areas and global impervious surface area.^42^

**Urban areas based on land cover**: A range of remote sensing projects identify ‘urban areas’ based exclusively on land cover classifications and building footprints, for example, the Global Urban Footprint surface,^43^ GlobCover, MODIS urban land cover, global land cover, among others.^42^

**Africapolis**: Defines an urban unit by combining satellite and aerial imagery, official demographic and other cartographic sources through a spatial approach. It applies a physical criterion (a continuously built-up area) and a demographic criterion (more than 10 000 inhabitants) to define an urban agglomeration.^30^

**NTL** from various sources has been either together with other datasets or as the main input to describe and define urban areas^19 42 44–46^ including predicting urban settlements and identify urbanisation rate, changes and boundaries.^19 42 44 46^

**Population**: Like NTL, population distribution has been used to define urbanisation either independently or in conjunction with additional datasets.^22 28 30 37 40 41^

**Auxiliary data**: Other approaches include the use of built environment, neighbourhood characteristics, style, and density of housing, share of commercial and agricultural activities, and access to services such as markets, communications, transportation, educational and health facilities and indicators often derived from census and household surveys.^11 17 24 25 31 37 47^

## DHS binary classes and GHS-Settlement Model layer-based urban gradient

**Figure 1:**
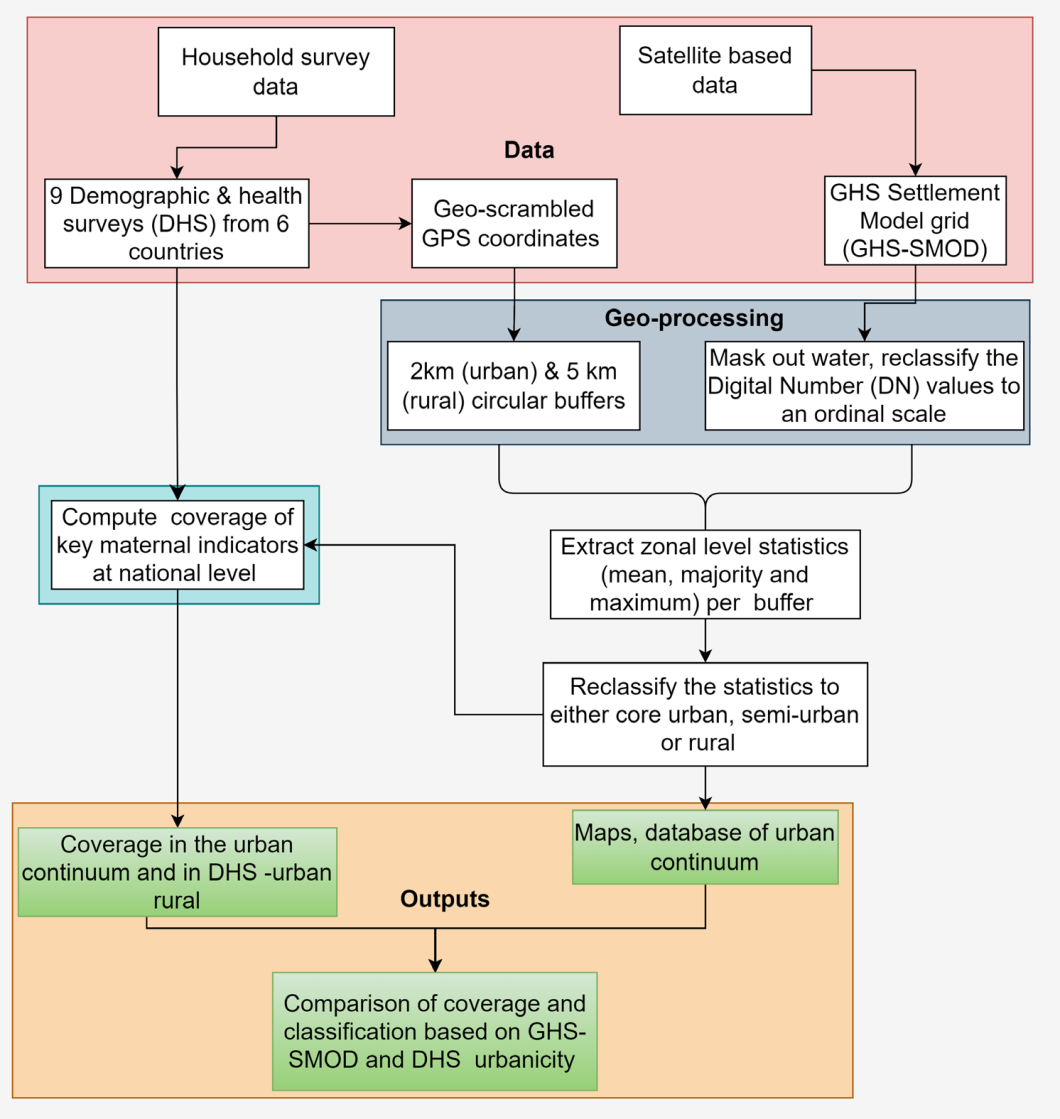
Flow chart of the steps adopted to reclassify Demographic and Health Survey (DHS) clusters to an urban continuum based on satellite-derived data.

To reclassify the DHS urban-rural classes to an urban gradient, we followed the geospatial methodology illustrated in figure 1 from our previous work on mainland Tanzania.^5^ The GHS-Settlement Model layer (GHS-SMOD) data consist of two levels: level 1 with three classes and level 2 with seven classes. To derive the urbanicity continuum, we used level 2 and not the three classes in level 1 for two reasons. First, the second hierarchical level has a high spatial granularity which discriminates between smaller settlements which are masked in level 1. Second, when investigating the mismatches between DHS and GHS-SMOD urbanicity classes, level 2 allows detecting subtle differences to better understand the dynamics of the mismatches. For example, one can pinpoint more accurately (with seven classes relative to 3) the characteristics of the nature of mismatched clusters. However, despite the advantages of working with seven classes, it presents challenges in terms of sample size for rare health outcomes such as mortality and other indicators common in DHS. Therefore, to overcome this challenge, we aggregated the seven classes into three categories, namely core urban, periurban and rural areas. This framework can also be used to derive more than three classes based on the nature of application and sample size limitations.

The initial step of the analysis involved masking out water from GHS-SMOD. Subsequently, after matching the DHS coordinates with the corresponding GHS-SMOD dataset, we reclassified the urbanicity classes into an ordinal scale, ranging from 1 (least urban) to 7 (most urban), to facilitate computations where each value has an attached logical meaning and interpretation. For each EA, we created a buffer zone (2 km for urban clusters and 5 km for rural clusters) and extracted the average, majority and maximum values using the zonal statistics of ArcMap V.10.5 (ESRI, Redlands, California, USA) within each buffer. The use of buffers helped mitigate the bias associated with the geoscrambling of cluster coordinates.^29^

After zonal statistics, the extracted values as expected ranged from 1 (very-low-density rural) to 7 (an urban centre) for the average, majority and maximum, and we input into our reclassification framework. A cluster was considered ‘*core-urban*’ if the mean, maximum and majority values within the buffer were at least 6. These values capture the urban centre grid and the dense urban cluster grid cell as defined in level 2.^33 34^ Where the mean values per buffer were ≥3 but less than 6, these were coded as ‘*semi-urban*’ capturing semidense urban cluster grid cell; sub-urban or periurban grid cell; and semidense, small clusters in the rural areas.^33 34^ Within the ‘*semi-urban*’ class, we also included values less than 3 but had a maximum of ≥4 to account for small towns surrounded by rural areas. Finally, all the remaining EAs were regarded as ‘*rural*’ and included mean value of ≤2, and the maximum values per buffer were less than 4. This encapsulated low-density and very-low-density rural grid cells.^33 34^ However, despite use of zonal statistics across buffered EAs, it is also possible that an EA originally located in a rural pixel could be misclassified as urban or semiurban based on these zonal statistics, and vice versa. The true location of the cluster remains uncertain given the geoscrambling of the coordinates that are publicly available from the DHS website.

**Figure 2:**
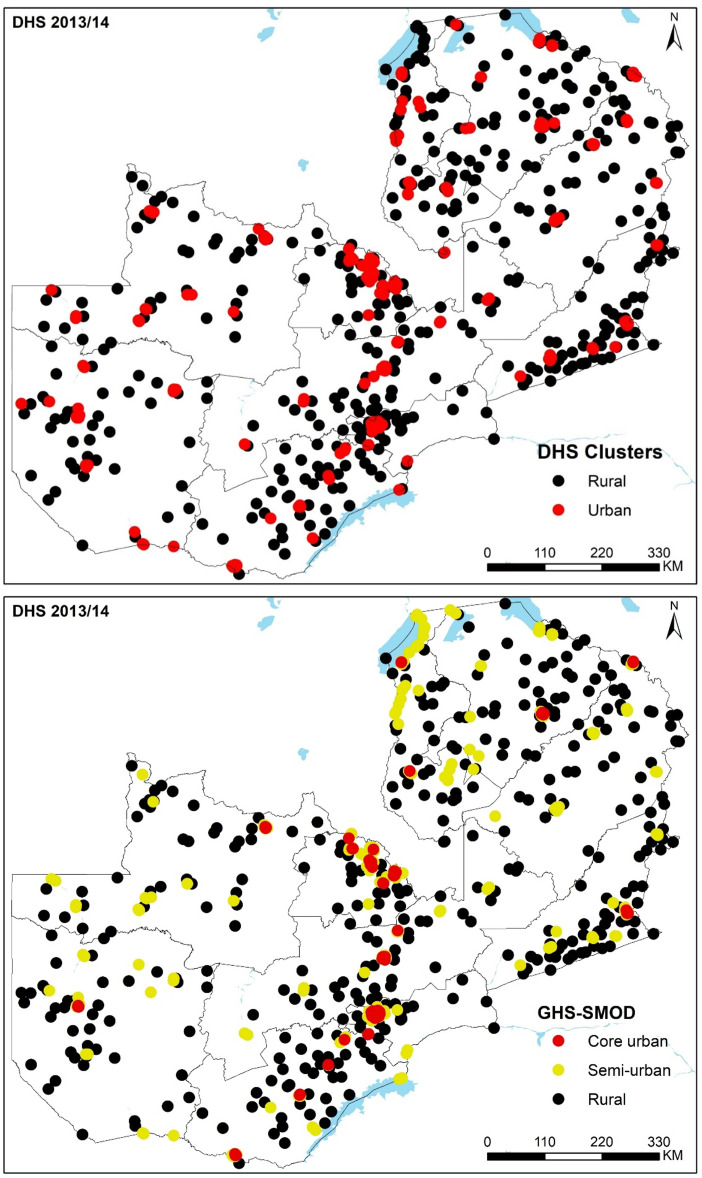
Seven hundred and nineteen clusters in the Zambia 2013/2014 Demographic and Health Survey (DHS) classified as urban and rural based on DHS clusters (upper panel) and as core urban, semiurban and rural based on 2015 GHS-Settlement Model layer (GHS-SMOD) gridded surface (lower panel).

The reclassified data for all six countries (DHS cluster and new urbanicity class for all nine surveys) have been made openly accessible in the Figshare repository.^35^ Here, we present the results for Zambia 2013/2014 DHS, while the results for the other eight surveys are found in the online supplemental data. A comparison with GHS-SMOD three-class urbanicity categories for Zambia shows that all 107 core urban areas were correctly classified as urban by the DHS. As expected, semiurban areas were a mix of urban (170) and rural (65) DHS classifications. It is worth noting that there were 28 EAs (7.4%) categorised as urban by the DHS, but GHS-SMOD classified them as rural (table 1 and figure 2). In the online supplemental file 1, we further interrogate EAs in Senanga and Kaoma towns, which were categorised as urban by the DHS and rural by GHS-SMOD. Similar results were obtained in the other countries (online supplemental data).

**Table 1:** DHS and GHS-SMOD classification of urbanicity based on Zambia 2013/2014 DHS clusters (n=719)

		GHS-SMOD urbanicity classes			
		Core urban	Semiurban	Rural	Total
DHS residence	Urban	107	170	28	305
	Rural	0	65	349	414
	Total	107	235	377	719

DHS, Demographic and Health Survey; GHS-SMOD, GHS-Settlement Model layer.

## Urban gradient and the coverage of key maternal health indicators

We computed the coverage (and 95% CIs) of nine maternal health indicators for Zambia 2013/2014 DHS, using both DHS urban-rural designation and three GHS-SMOD urbanicity categories (online supplemental data 1). The multilevel cluster survey design used in the DHS was accounted for in Stata (V.15.0, Stata, College Station, Texas). The amount of variability in the coverage estimates of the health indicators is related to the misalignment between the urbanity classes in the DHS and the GHS-SMOD. Therefore, in addition, to explore any potential differences between countries, similar coverage estimates were computed for Ghana (DHS 2014) and Ethiopia (DHS 2019). Four insights can be derived from this comparison (table 2).

**Table 2:** Coverage of key maternal health indicators by DHS binary urban/rural classes and GHS-SMOD urban gradient for Zambia 2013/2014, Ghana 2014 and Ethiopia 2019 DHS, % (95% CI)

Zambia 2013/2014		DHS		GHS-SMOD		
Category	Indicator	Urban	Rural	Core urban	Semiurban	Rural
Modern contraceptive prevalence		53.4 (51.8–55.0)	39.0 (37.7–40.2)	53.7 (51.7–55.8)	**48.5 (46.5–50.5)**	39.3 (38.0–40.6)
Healthcare access problems	Permission to go	2.1 (1.8–2.4)	3.3 (3.0–3.7)	2.2 (1.8–2.7)	2.2 (1.8–2.7)	3.4 (3.0–3.8)
	Getting money	11.9 (11.1–12.6)	34.8 (33.8–35.8)	10.4 (9.5–11.4)	**16.9 (15.8–18.1)**	35.7 (34.6–36.8)
	Distance to facility	**12.9 (12.2–13.7)**	57.0 (56.0–58.1)	**10.8 (9.9–11.8)**	**23.6 (22.3–24.8)**	57.9 (56.8–59.0)
	Unable to go alone	7.2 (6.7–7.8)	25.6 (24.7–26.6)	7.0 (6.3–7.8)	**9.8 (9.0–10.7)**	26.7 (25.7–27.7)
No antenatal care (ANC)		0.9 (0.6–1.2)	1.7 (1.4–2.0)	0.4 (0.2–0.9)	1.5 (1.0–2.0)	1.7 (1.3–2.0)
Place of childbirth	Health facility	**89.2 (88.2–90.0)**	56.5 (55.5–57.6)	**91.9 (90.8–93.0)**	**79.1 (77.7–80.5)**	55.6 (54.5–56.7)
	Home	**10.7 (9.8–11.6)**	42.1 (41.1–43.2)	**8.0 (7.0–9.1)**	**20.6 (19.2–22.0)**	42.9 (41.8–44.0)
	Others	0.2 (0.1–0.3)	1.4 (1.1–1.6)	0.1 (0.0–0.3)	0.3 (0.1–0.5)	1.5 (1.2–1.8)
Sector of facility for childbirth	Public	**84.6 (83.5–85.6)**	51.7 (50.7–52.7)	**87.8 (86.5–89.1)**	**73.2 (71.6–74.7)**	51.2 (50.0–52.3)
	Private	4.6 (4.0–5.2)	4.8 (4.4–5.3)	4.1 (3.4–5.0)	5.9 (5.1–6.8)	4.5 (4.0–4.9)
	Home	**10.7 (9.8–11.6)**	42.1 (41.1–43.2)	**8.0 (7.0–9.1)**	**20.6 (19.2–22.0)**	42.9 (41.8–44.0)
	Others	0.2 (0.1–0.3)	1.4 (1.1–1.6)	0.1 (0.0–0.3)	0.3 (0.1–0.5)	1.5 (1.2–1.8)
Caesarean section rate		7.2 (6.5–8.0)	3.0 (2.6–3.3)	8.3 (7.2–9.4)	**4.9 (4.2–5.7)**	3.0 (2.7–3.4)
**Ghana 2014**						
Modern contraceptive prevalence		19.8 (18.3–21.4)	25.0 (23.4–26.7)	19.4 (17.6–21.4)	22.0 (20.3–23.8)	26.4 (24.2–28.8)
Healthcare access problems	Permission to go	5.5 (4.9–6.2)	6.6 (5.9–7.4)	4.6 (3.9–5.4)	6.3 (5.6–7.2)	7.7 (6.6–8.8)
	Getting money	**35.2 (33.9–36.5)**	49.4 (47.9–50.9)	**30.1 (28.6–31.7)**	**45.1 (43.5–46.6)**	52.9 (50.9–55.0)
	Distance to facility	**17.2 (16.2–18.3)**	**34.9 (33.3–36.1)**	**14.1 (13.0–15.4)**	**26.9 (25.5–28.3)**	**38.5 (36.5–40.5)**
	Unable to go alone	13.7 (12.7–14.6)	17.9 (16.8–19.1)	12.9 (11.7–14.0)	15.4 (14.3–16.6)	20.0 (18.4–21.7)
No ANC		1.4 (0.92–2.0)	3.7 (3.0–4.5)	1.2 (0.68–2.0)	2.2 (1.6–3.1)	4.4 (3.4–5.7)
Place of childbirth	Health facility	**90.3 (89.1–91.4)**	**59.3 (57.5–61.1)**	**94.4 (93.1–95.4)**	**74.4 (72.6–76.2)**	**53.8 (51.5–56.1)**
	Home	**9.4 (8.3–10.6)**	**40.3 (38.6–42.1)**	**5.4 (4.3–6.6)**	**25.3 (23.6–27.2)**	**45.7 (43.4–48.0)**
	Others	0.32 (0.16–0.63)	0.36 (0.20–0.65)	0.28 (0.11–0.72)	0.23 (0.10–0.53)	0.54 (0.29–1.01)
Sector of facility for childbirth	Public	76.5 (74.8–78.1)	**55.9 (54.1–57.6)**	77.9 (75.7–80.0)	**67.8 (65.9–69.7)**	**51.0 (48.7–53.3)**
	Private	13.8 (12.5–15.2)	3.4 (2.8–4.1)	16.5 (14.8–18.5)	**6.6 (5.7–7.7)**	2.7 (2.1–3.6)
	Home	**9.4 (8.3–10.6)**	**40.3 (38.6–42.1)**	**5.4 (4.3–6.6)**	**25.3 (23.6–27.2)**	**45.7 (43.4–48.0)**
	Others	0.32 (0.16–0.63)	0.36 (0.20–0.65)	0.28 (0.11–0.72)	0.23 (0.10–0.53)	0.54 (0.29–1.01)
Caesarean section rate		18.7 (17.3–20.3)	8.0 (7.1–9.0)	21.7 (19.7–23.8)	**12.1 (10.8–13.5)**	6.3 (5.2–7.5)
**Ethiopia 2019**						
Modern contraceptive prevalence		47.7 (45.3–50.1)	**37.6 (36.2–39.1)**	47.1 (42.2–52.0)	44.3 (42.7–46.0)	**32.1 (30.0–34.2)**
No ANC		**15.2 (13.2–17.6)**	29.4 (27.7–31.1)	**2.7 (1.2–5.8)**	**24.1 (22.4–25.9)**	32.0 (29.6–34.4)
Place of childbirth	Health facility	**70.4 (67.9–72.7)**	40.2 (38.8–41.7)	**96.3 (93.4–98.8)**	**50.0 (48.1–51.6)**	37.9 (35.9–40.0)
	Home	**29.2 (26.9–31.7)**	58.5 (57.0–60.0)	**3.5 (1.9–6.3)**	**49.1 (47.3–50.8)**	61.0 (58.9–63.1)
	Others	0.41 (0.18–0.93)	1.2 (0.95–1.6)	0.22 (0.02–2.5)	1.1 (0.77–1.5)	1.1 (0.73–1.6)
Sector of facility for childbirth	Public	**63.2 (60.6–65.7)**	39.7 (38.2–41.2)	**74.7 (69.3–79.3)**	**48.3 (46.6–50.1)**	37.3 (35.3–39.4)
	Private	**7.2 (5.9–8.7)**	0.58 (0.39–0.86)	**21.7 (17.3–26.8)**	**1.5 (1.1–2.0)**	0.59 (0.17–1.0)
	Home	**29.2 (26.9–31.7)**	58.5 (57.0–60.0)	**3.5 (1.9–6.3)**	**49.1 (47.3–50.8)**	61.0 (58.9–63.1)
	Others	0.41 (0.18–0.93)	1.2 (0.95–1.6)	0.22 (0.02–2.5)	1.1 (0.77–1.5)	1.1 (0.73–1.6)
Caesarean section rate		**10.1 (8.6–11.9)**	3.9 (3.3–4.5)	**21.6 (17.2–26.7)**	**5.5 (4.8–6.4)**	3.2 (2.5–4.0)

Indicators under the category of *healthcare access problems* were not available in Ethiopia DHS 2019.

Bold : Rural EAs have different estimates between DHS and GHS-SMOD or where DHS; DHS urban EAs have different estimates to those of GHS-SMOD core urban or semi-urban.

DHS, Demographic and Health Survey; GHS-SMOD, GHS-Settlement Model layer.

First, all is not equal within urban areas. For example, the percentage of births occurring at home in semiurban areas is more than twice those in the core urban areas. Therefore, if the government was to intervene, different strategies and resources may be required for these two diverse types of urban areas. These semiurban areas are expanding and mainly characterised by low-income households^18 23 24^ but also exhibit numerous functional urban attributes at the edge of the core urban areas.^28^ Related to the variation within urban areas is effective targeting of hot spots. Consider the distance to facility which seems to be a lesser of problem in the core urban (10.8%) relative to the newly defined semiurban (23.6%) in Zambia. The semiurban class can be prioritised and targeted with unique interventions instead of a blanket approach in both urban and semiurban given the limited resources.

Second, there seems to be less variability between coverage in the rural areas (based on DHS and GHS-SMOD). For example, the coverage of births at a health facility was 56.5% and 55.6%, respectively. However, there are some differences in urban and core urban as defined by the DHS and GHS-SMOD (table 2). Where the rural EAs have different estimates between DHS and GHS-SMOD (based on 95% CI), they are highlighted in bold (table 2). The same analogy has been applied to urban areas. Further, unsurprisingly, differences between estimates of maternal health indicators in urban areas (DHS) and semiurban areas (GHS-SMOD) were clear in most of the indicators (table 2, semiurban estimate in bold).

Third, while majority of the indicators showed linear relationships (table 2), there was evidence of non-linear relationship when looking at the urban continuum which was not evident in the DHS urban-rural dichotomy. In the case of Zambia (table 2), the childbirths occurring at the private sector depict a reversed arch shape (∩), where the rates are high in periurban areas and lower in both core urban and rural areas. The continuum allowed this type of relationship between urbanicity and health outcomes to be appreciated and is inherently masked in the DHS’s two classes of urban and rural. Finally, the consistent definition of urban, semiurban and rural areas facilitates a fair comparison of the maternal health indicators. For example, the caesarean rates in core urban areas were similar in Ghana (21.7 (19.7–23.8)) and Ethiopia (21.6 (17.2–26.7)), more than two times the rates in Zambia (8.3 (7.2–9.4)).

## Strengths and limitations

The three urbanicity categories allow for the differentiation of semiurban areas from core, densely populated inner cities, and retain a third category of rural areas. Overall, there are two main advantages of the GHS-SMOD expanded categories: first, the ability to disaggregate semiurban areas from urban areas, and second, the ability to correct misalignment in administrative/historical designation of areas which appear to be urban on the DHS but are in fact more rural in character.

Related reclassification analysis using other urbanicity datasets (box 1) and subsequent comparison have been conducted with applications in safety of drinking water,^28^ coastal hazard risk^36^ and understanding the utility of urbanisation covariates.^14^ Among these studies, Dorélien and colleagues compared the urban-rural classification derived from Global Rural-Urban Mapping Project with those used by countries in the DHS; however, while we considered urbanicity as a gradient, they approached it from a binary perspective.^14^

Our analysis was constrained by the temporal resolution of the GHS-SMOD dataset, which is available every 5 years. This means that some surveys may have a time difference of at least 2.5 years from the temporally closest GHS-SMOD data, potentially leading to some inaccuracies. For example, if the survey was conducted in mid-2017, the choice of GHS-SMOD dataset will be either 2015 or 2020. Therefore, the urbanicity in mid-2017 is proxied by those data from 2020 or 2015 which may result in inaccuracies especially in the face of rapid urbanisation. Further, GHS-SMOD dataset is derived from the population and built-up areas based on the most accurate and up-to-date 2023 version. However, the accuracy of the GHS-SMOD layer will depend on these two inputs. The population grids are based on census data (often available for large subnational areas course resolution) augmented with covariates which are products of modelling and remote sensing. The built-up layer from satellite imagery may fail to detect all built-up areas or may erroneously identify some areas as built-up or as covered by a building.

Despite accounting for the displacement of DHS clusters through buffers, our results may still have residual errors due to the geoscrambling of DHS cluster coordinates. This is because the exact location of the cluster could have been anywhere within the buffer and the true location will remain unknown to the public to preserve the confidentiality and privacy of the survey respondents. While it was pragmatic and logical to use the mean and the majority values in deriving the urbanicity classes, other approaches may lead to different results relative to those reported in this analysis.

We categorised our data into three classes to capture the variations within urban areas. However, like the inclusion of semiurban classes, it could be argued that we should have included a *semirural* category^22^ or even explore the heterogeneity within urban areas further.^11^ Our approach allows for this expansion; however, due to the sample size of the sampled EAs in the DHS, for this case study we present three categories only. Further, to better understand within-urban or semiurban variation and inequalities, additional data and analysis will be required to discriminate between slums and non-slums within core urban, areas of conflict within the periurban or the urban poor and urban rich.

## Conclusion

Definitions that more accurately capture urban-rural continuum in the context of population health have never been more crucial. There is ongoing rapid urbanisation,^1^ reversal and shrinking of the urban advantage^5^ and increasing heterogeneity and inequalities within the urban areas.^9^ The dichotomous and crude classification of urban and rural areas lacks specificity in predicting health outcomes^24^ and is characterised by infrequent updates which all may lead to bias.^20 25 37^ Satellite-derived data augmented with population density and built-up areas can provide comparable, objective and continuous measures of urban gradient which together more accurately and timely quantify the extent of urbanisation at a high spatial resolution, including variations *within* urban areas.

Researchers and stakeholders working on population health in urban settings should complement national statistical offices’ definitions of urban areas by incorporating alternative data, such as satellite imagery combined with other auxiliary datasets, to facilitate a comprehensive analysis of urbanisation and health outcomes. For example, Liberia (and Cameroon) in their recent DHS disaggregated the urban category to contain Greater Monrovia and other urban. Calculating an estimate based on existing urban-rural class followed by a subcategory based on satellite could be a starting point. The task of defining the urban continuum remains an ongoing challenge that requires attention considering population health.

We argue for a critical reflection and dialogue when health indicators are being disaggregated by urban gradient. For example, when historical trends of health indicators are to be computed based on DHS data, historical satellite imagery is available for retrospective analyses and prospective scenarios. Such urbanicity data can also be integrated with the routine health information systems where indicators require disaggregation. However, for integration, technical expertise would be required. We hope the database we have provided will be a useful starting point and such analyses will be conducted in other countries where household survey data (DHS and MICS) are available. Second, while we recognise that countries also use these urban-rural labels for other purposes beyond population health, our analysis was focused on implications for population health and in the context of household survey data. Potentially, countries could opt to use satellite imagery-based classification in relation to population health and other administrative definitions for political and related purposes.

## Data Availability

Data are available in a public, open access repository. Data are available in a public, open access repository. DHS datasets are available from the DHS programme upon request at https://dhsprogram.com/data/available-datasets.cfm. The urbanicity surfaces can be accessed at https://ghsl.jrc.ec.europa.eu/download.php?ds=smod while the reclassified urban classes for the six countries are available at the Figshare repository at https://doi.org/10.6084/m9.figshare.23559225.
