# Supplementary material for: Exploring the urban gradient in population health: insights from satellite-derived urbanicity classes across multiple countries and years in sub-Saharan Africa
Source: BMJ Glob Health. 2023 Oct 21;8(10):e013471. doi: 10.1136/bmjgh-2023-013471 (PMC10603412; doi:10.1136/bmjgh-2023-013471)
Supplement: Supplementary data [file bmjgh-2023-013471supp001.pdf]

Data Supplement

Definition of urban areas

Table 1: Definitions of urban areas in six selected African countries that are often used by the Demographic and Health Survey (DHS) program to delineate between urban and rural enumeration areas during data collection.

ID	Country	Country-specific definition of urban area adopted by DHS
1	Cameroon	Administrative centres of territorial units (district, subdivision, division, or province) or any locality with 5,000 inhabitants or more and with sufficient socioeconomic and administrative infrastructures [1]
2	Ethiopia	Localities with 2,000 inhabitants or more [1]
3	Ghana	Any settlement with a population of 5,000 or more persons [2]
4	Guinea	Administrative centres or prefectures [1]
5	Kenya	An area with a high density of people and human-created structures and which has a total population of at least 2,000 people including cities, municipalities, town councils, and urban councils [3]
6	Zambia	A locality with ≥ 5,000 people, 50% of whom are not engaged in agriculture and should have urban attributes such as piped water, and electricity. If a centre has < 5000 people but has these urban characteristics, it is classified as urban. Also considered urban is district and provincial administrative centres [4].

Ghana

The 2014 Ghana DHS with 423 clusters that had coordinates were matched with 2015 GHS-SMOD gridded surface. The DHS clusters were equally distributed in the urban (207-48.9%) and rural areas (216-51.1%) (*Figure 1*).

Figure 1: 423 clusters in the 2014 Ghana DHS with coordinates

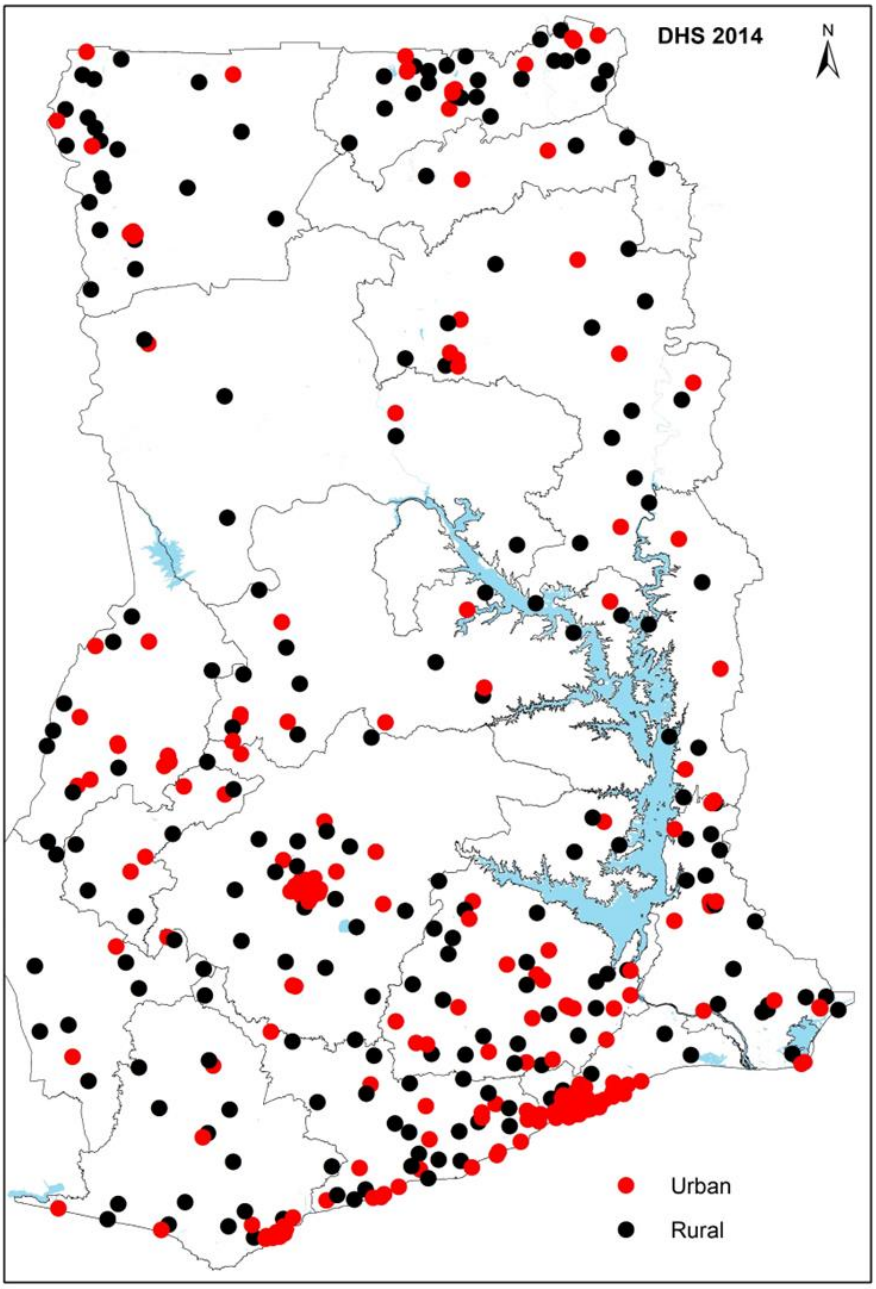

The GHS-SMOD urbanicity surface is shown in *Figure 2* (left panel) along with zoomed-in views of key urban areas like Accra, Kumasi, Tumale, Wa, and Sekondi Takoradi. *Figure 2* (right panel) displays the reclassification results, indicating three classes: core urban (113 clusters), semi-urban (192 clusters), and rural (188 clusters). A comparison between GHS-SMOD and DHS clusters revealed overall agreement (*Table 2*). Specifically, the semi-urban category is expected to encompass both urban and rural clusters from the DHS data. The DHS classified all core-urban clusters as urban, except for one cluster. Additionally, all GHS-SMOD rural clusters were classified as rural by the DHS, except for four clusters labelled as urban

Figure 2: Ghana 2015- GHS-SMOD urbanization surface (left panel) and corresponding derived clusters (right panel)

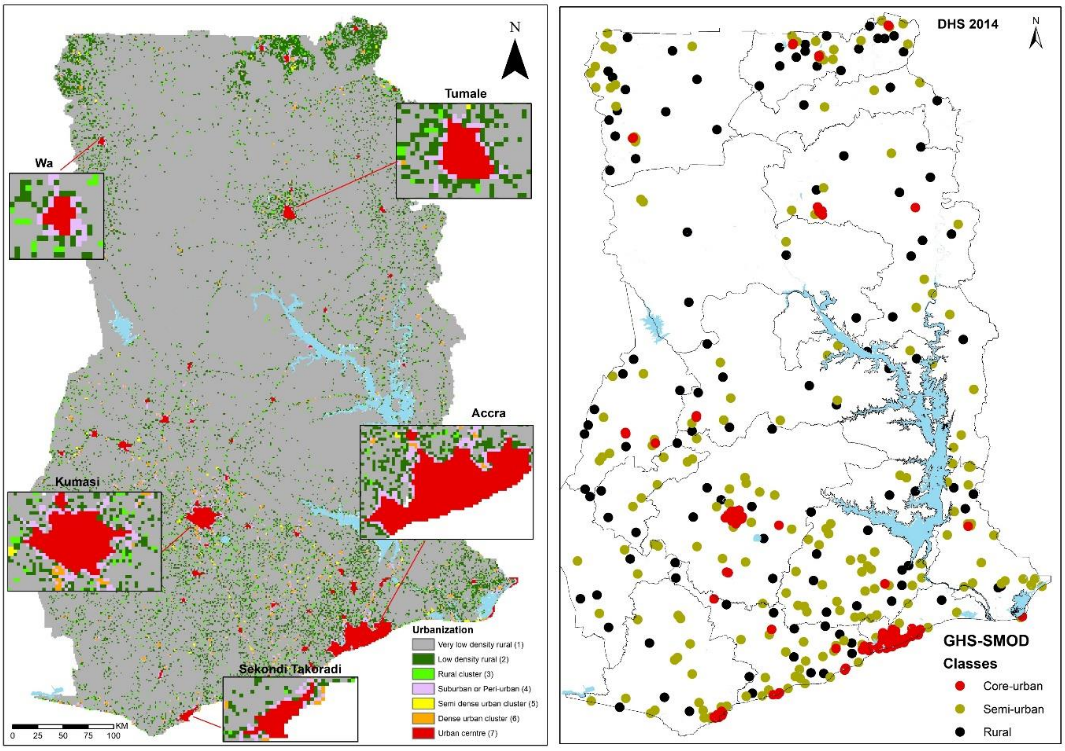

Table 2: DHS and GHS-SMOD classification of urbanicity in Ghana based on DHS 2014 clusters

		GHS-SMOD urbanicity classes			
		Core urban	Semi urban	Rural	Total
**DHS residence**	Urban	112	100	4	207
	Rural	1	92	114	216
	Total	113	192	118	423

Guinea

Guinea 2005 DHS had 291 clusters with coordinates which were matched with GHS-SMOD 2005. Figure 3 shows the spatial distribution the 219 clusters with geographical coordinates across Guinea. About a third (34.4%, n=100) of these clusters were in the urban areas and the rest were as urban (n=191).

Figure 3: 219 clusters that had geographical coordinates in the Guinea's 2005 DHS

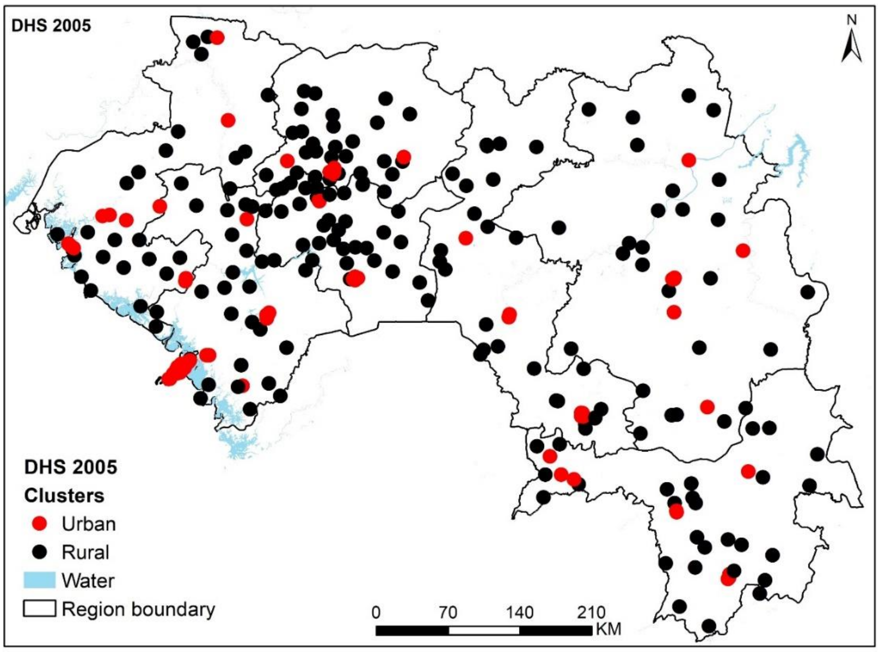

The GHS-SMOD urbanicity surface is shown in Figure 4 and some of the main urban areas zoomed in, including Conakry, Kindia, Kankan, Nzerekore and Labe. The reclassification results are shown in Figure 5 with 3 classes, core urban (54 clusters), semi-urban (78 clusters) and rural (159 clusters). When the GHS-SMOD and DHS clusters spatially overlaid, there was an overall agreement (Table 3: DHS versus GHS-SMOD classification of urbanicity based on Guinea's 2005 DHSTable 3). That is, semi urban category would be expected to have both urban and rural clusters from the DHS. All core-urban clusters were classified as urban by DHS except one. All GHS-SMOD rural clusters were classified as rural by DHS except nine which had been classified as urban in the DHS.

Table 3: DHS versus GHS-SMOD classification of urbanicity based on Guinea's 2005 DHS

		GHS-SMOD urbanicity classes			
		Core urban	Semi urban	Rural	Total
**DHS residence**	Urban	53	38	9	100
	Rural	1	40	150	191

Total	54	78	159	291

Figure 4: Gridded urbanization classes of GHS-SMOD 2005 in Guinea

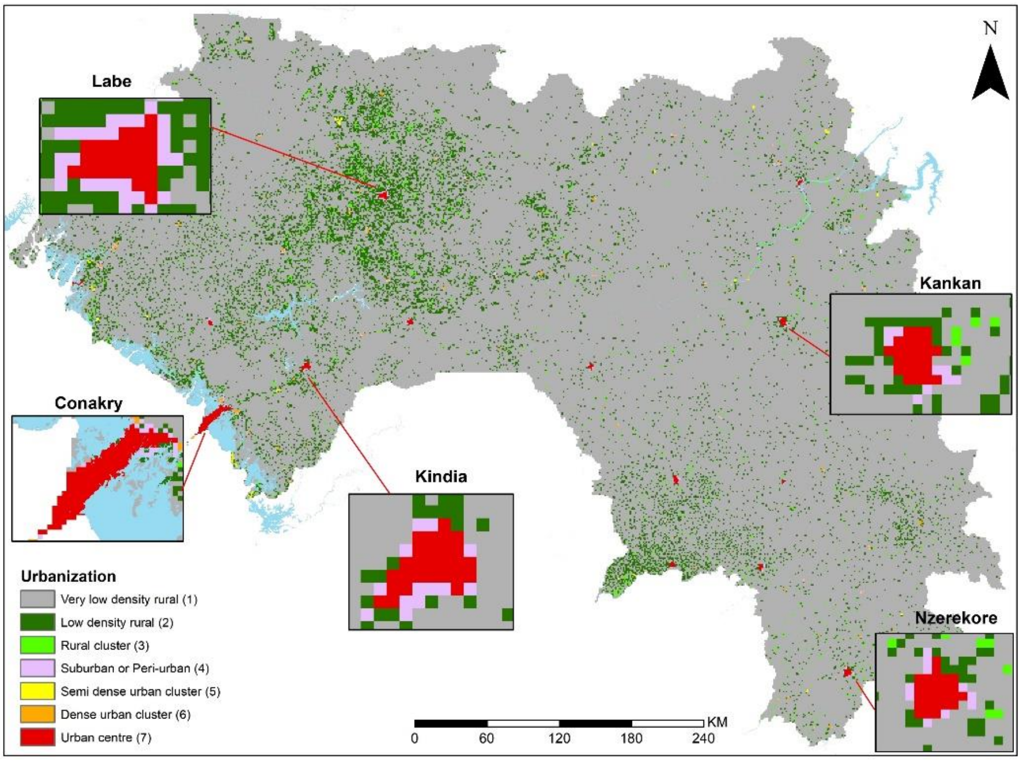

Figure 5: Urbanization classes based on GHS-SMOD 2005 per 2005 DHS cluster in Guinea

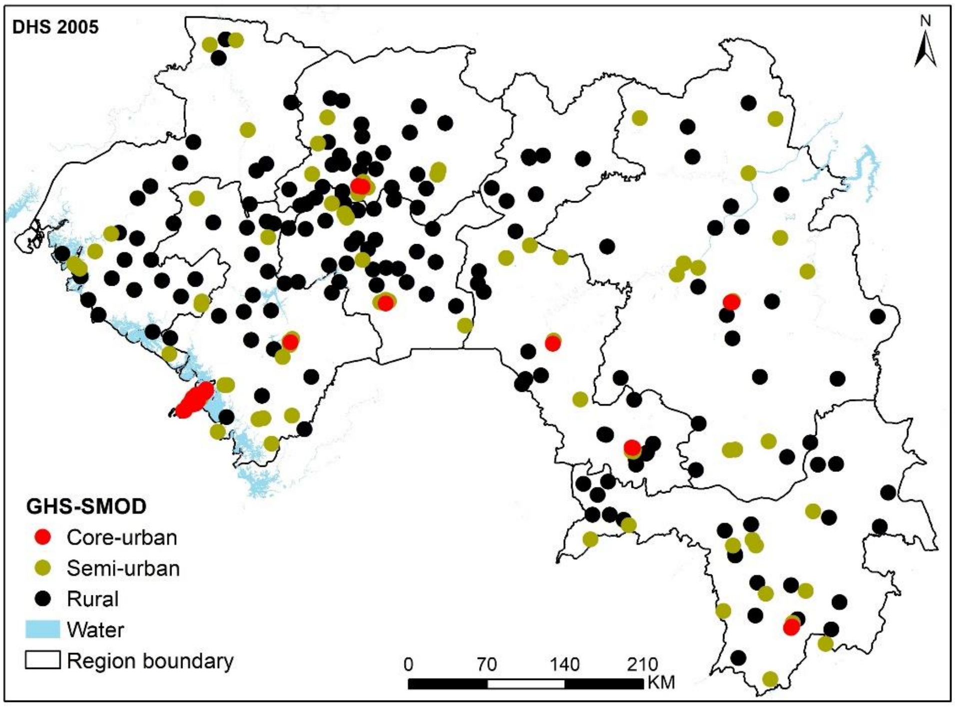

Kenya

Two surveys were considered in Kenya, **DHS 2008/09** (Figure 6, left panel) with an overall 397 clusters after excluding one with a missing coordinate and **DHS 2014** (Figure 6, right panel) with 1585 clusters, exclusive of 9 clusters without coordinates. These two surveys were matched to GHS-SMOD surfaces from 2010 and 2015, respectively.

Figure 6: Clusters that had geographical coordinates in Kenya based on DHS2008/09 (397 clusters) and DHS 2014 (1585 clusters).

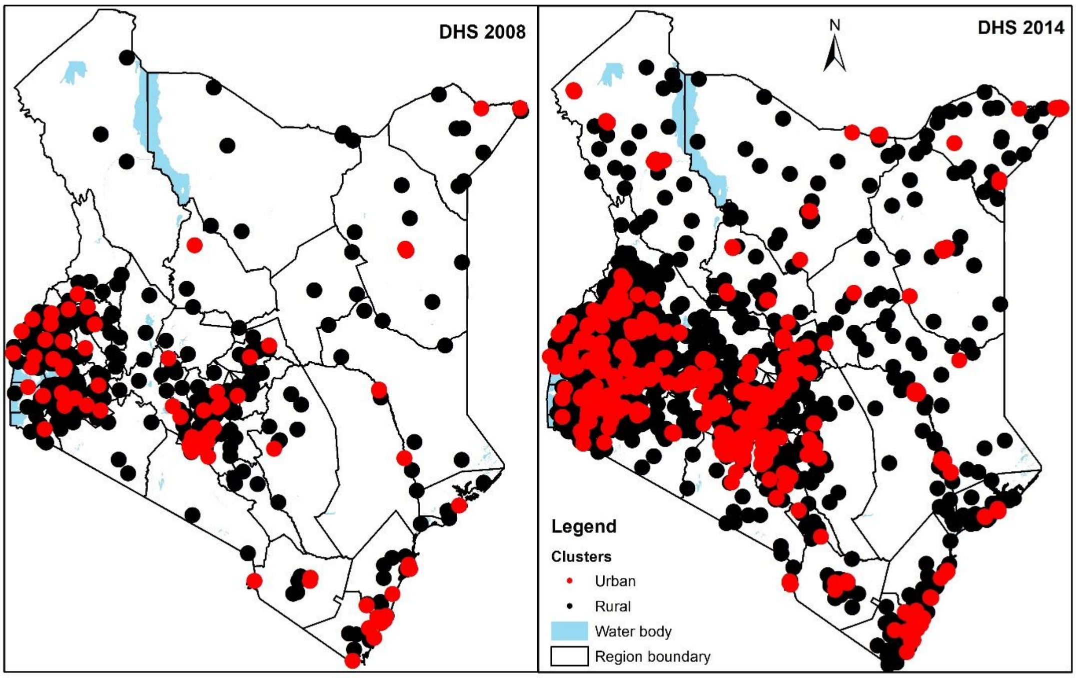

The GHS-SMOD urbanicity surfaces for the two years are shown in Figure 7 and some of the main urban areas highlighted including Nairobi, Nakuru, Mombasa, Kisumu and Eldoret. The reclassification results are shown in Figure 8 with 3 classes, core urban, semi-urban and rural. When the GHS-SMOD and DHS clusters spatially overlaid, the results are shown in Table 3: DHS versus GHS-SMOD classification of urbanicity based on Guinea's 2005 DHSTable 4 and Table 5 for the two surveys, respectively. Semi urban category had majority of the clusters in both surveys, while the urban and rural categories followed similar patterns as Ghana and Guinea.

Figure 7: GHS-SMOD gridded surfaces for Kenya, 2010 (left panel) and 2015 (right panel). The major urban areas are also shown

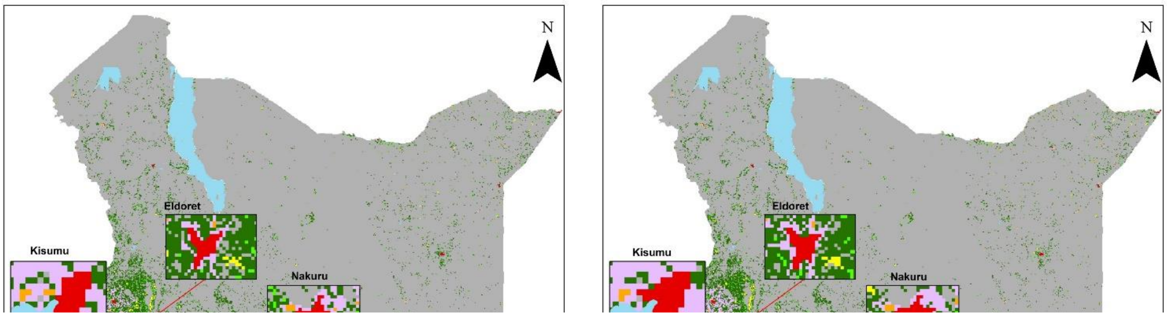

Figure 8: Three classes of urbanization defined from GHS-SMOD data for 2010 and 2015 based on DHS 2008/09 and DHS 2014 surveys in Kenya, respectively

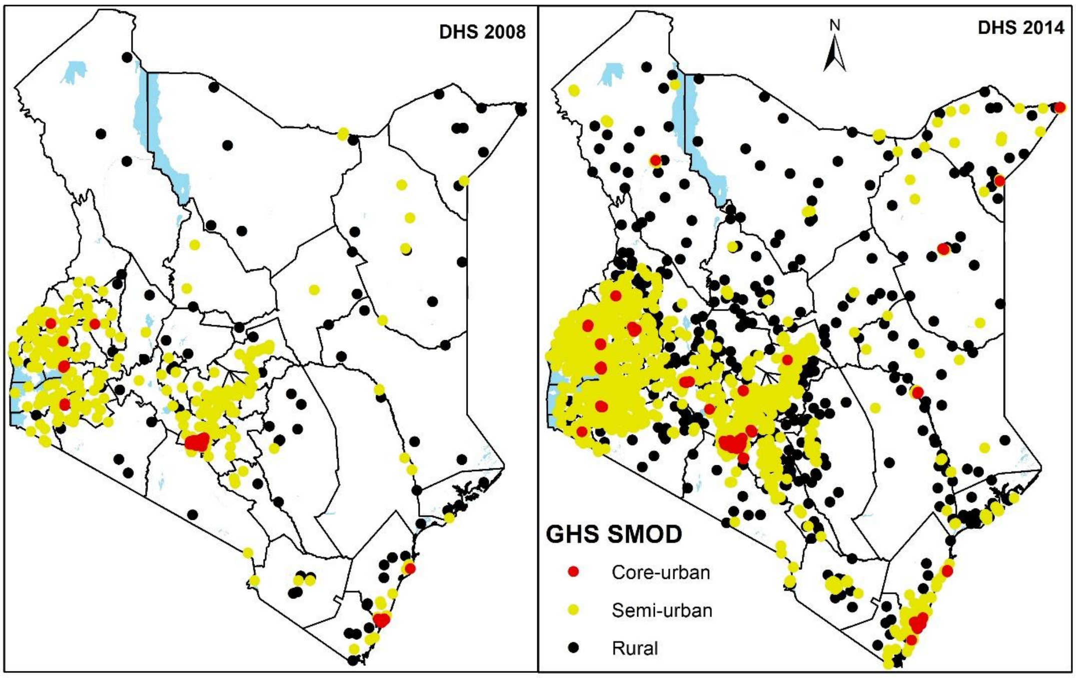

Table 4: DHS and GHS-SMOD classification of urbanicity based on Kenya's 2008/09 DHS clusters

		GHS-SMOD urbanicity classes- DHS 2008/09			
		Core urban	Semi urban	Rural	Total

**DHS residence**	Urban	60	64	8	265
	Rural	1	193	71	132
	Total	61	257	79	397

Table 5: DHS and GHS-SMOD classification of urbanicity based on Kenya's 2014 DHS clusters

		GHS-SMOD urbanicity classes- DHS 2014			
		Core urban	Semi urban	Rural	Total
**DHS residence**	Urban	157	434	34	615
	Rural	0	676	294	970
	Total	157	1100	328	1585

Ethiopia

Two surveys were considered in Ethiopia, **DHS 2016** (Figure 9, left panel) with an overall 622 clusters after excluding 21 clusters with missing coordinates and **DHS 2019** (Figure 9, right panel) with 305 clusters. These two surveys were matched to GHS-SMOD surfaces from 2015 and 2020, respectively.

Figure 9: Clusters that had geographical coordinates in Ethiopia based on DHS2016 (622 clusters) and DHS 2019 (305 clusters).

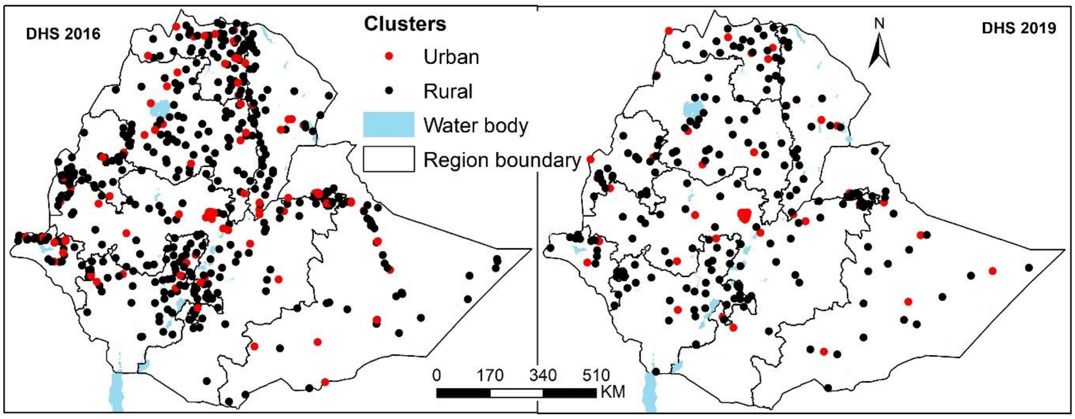

The GHS-SMOD urbanicity surfaces for the two years are shown in Figure 10 and some of the main urban areas highlighted including Addis Ababa, Mekele, Bahir Dar, Hawasa and Gambela. The reclassification results are shown in Figure 11 with 3 classes, core urban, semi-urban and rural. When the GHS-SMOD and DHS clusters spatially overlaid, the results are shown in Table 3: DHS versus GHS-SMOD classification of urbanicity based on Guinea's 2005 DHSTable 6 and Table 7 for the two surveys, respectively. Semi urban category had majority of the clusters in both surveys, while the urban and rural categories followed similar patterns as Kenya, Ghana and Guinea.

Figure 10: GHS-SMOD gridded surfaces for Ethiopia, 2015 (left panel) and 2020 (right panel). The major urban areas are also shown

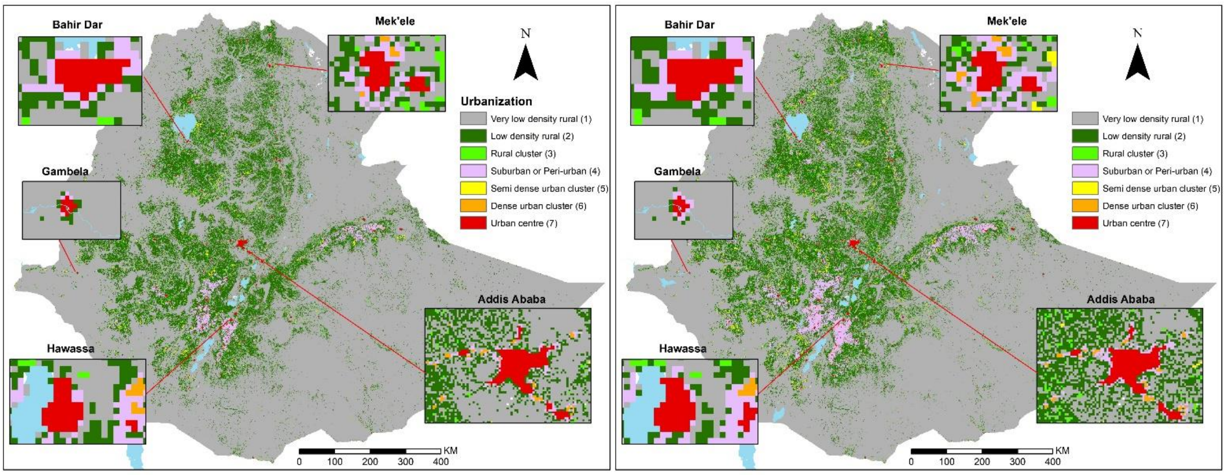

Figure 11: Three classes of urbanization defined from GHS-SMOD data for 2015 and 2020 based on DHS 2016 and DHS 2020 surveys in Ethiopia, respectively

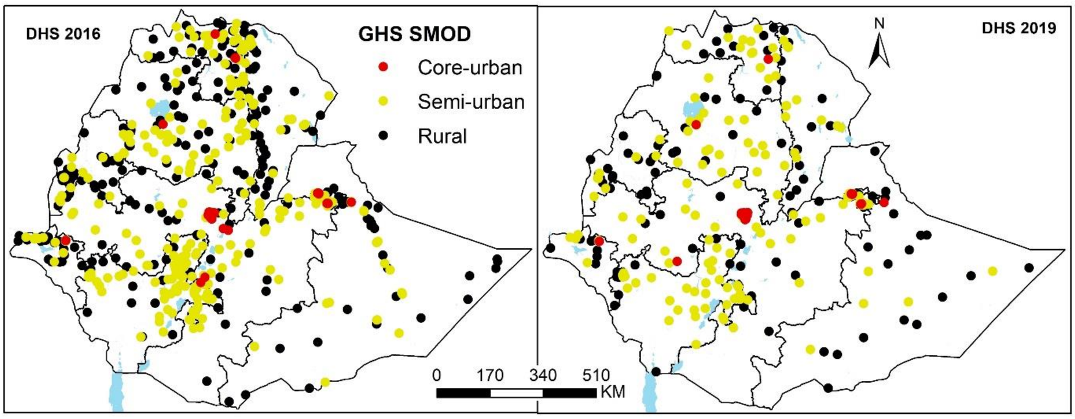

Table 6: DHS and GHS-SMOD classification of urbanicity based on Ethiopia's 2016 DHS clusters

		GHS-SMOD urbanicity classes- DHS 2016			
		Core urban	Semi urban	Rural	Total
**DHS residence**	Urban	90	109	3	202
	Rural	0	200	220	420
	Total	90	309	223	622

Table 7: DHS and GHS-SMOD classification of urbanicity based on Ethiopia's 2019 DHS clusters

		GHS-SMOD urbanicity classes- DHS 2019			
		Core urban	Semi urban	Rural	Total
**DHS residence**	Urban	40	46	7	93
	Rural	0	113	99	212
	Total	40	259	106	305

Cameroon

Two surveys were considered in Cameroon, **DHS 2004** (Figure 12, left panel) with an overall 464clusters after excluding 2 clusters with missing coordinates and **DHS 2011** (Figure 12, right panel) with an overall 577 clusters, exclusive of one cluster without coordinates. These two surveys were matched to GHS-SMOD surfaces from 2005 and 2010, respectively.

Figure 12: Clusters that had geographical coordinates in Cameroon based on DHS 2004 (464 clusters) and DHS 2011 (577 clusters).

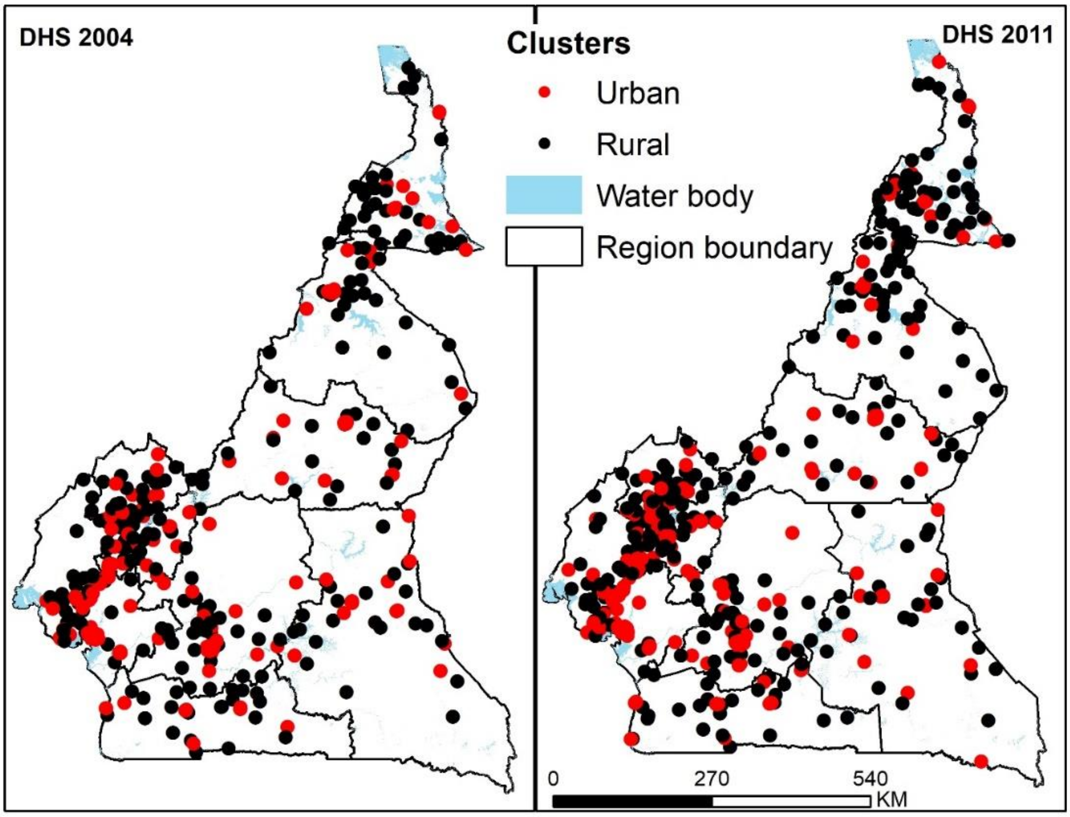

The GHS-SMOD urbanicity surfaces for the two years are shown in Figure 13 and some of the main urban areas highlighted including Yaounde, Doula, Ngaoundure, Garoua and Bamenda. The reclassification results are shown in Figure 14 with 3 classes, core urban, semi-urban and rural. When the GHS-SMOD and DHS clusters spatially overlaid, the results are shown in Table 3: DHS versus GHS-SMOD classification of urbanicity based on Guinea's 2005 DHSTable 8 and Table 9 for the two surveys, respectively. The patterns, overall, were like those of Ethiopia Kenya, Ghana, and Guinea.

Figure 13: GHS-SMOD gridded surfaces for Cameroon, 2005 (left panel) and 2010 (right panel). The major urban areas are also shown

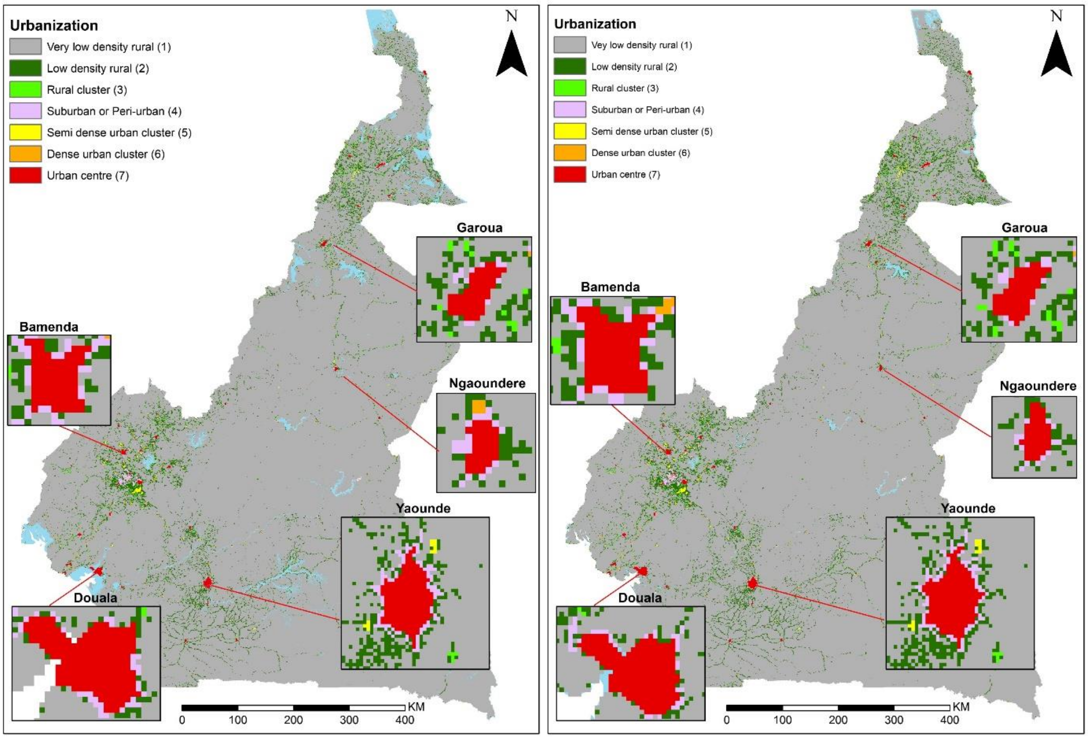

Figure 14: Three classes of urbanization defined from GHS-SMOD data for 2005 and 2011 based on DHS 2004and DHS 2011 surveys in Cameroon, respectively.

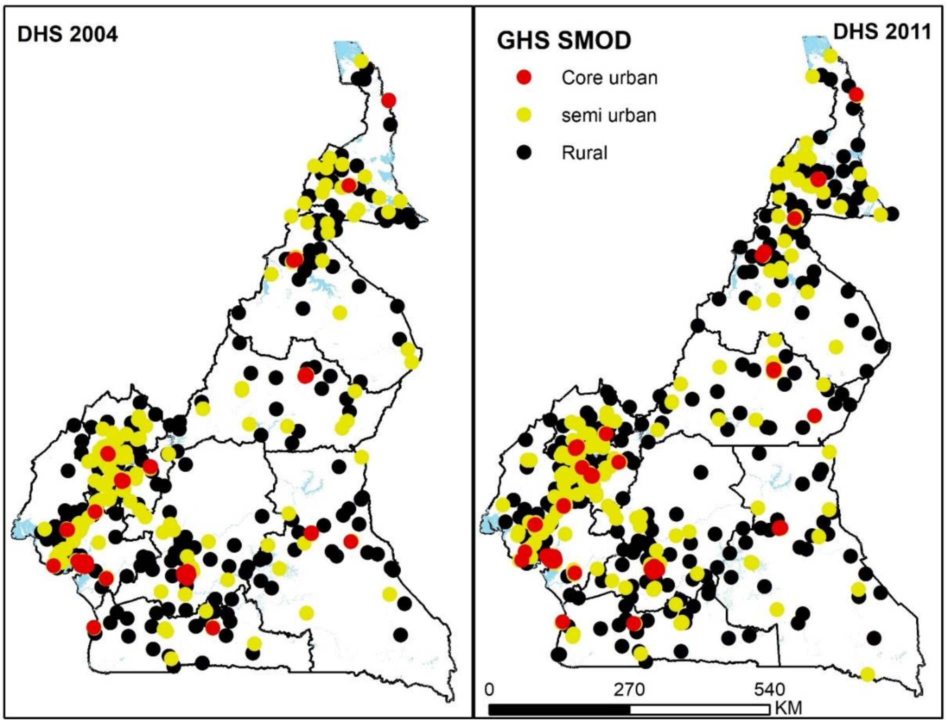

Table 8: DHS and GHS-SMOD classification of urbanicity based on Cameroon's 2004 DHS clusters

		GHS-SMOD urbanicity classes- DHS 2004			
		Core urban	Semi urban	Rural	Total
DHS residence	Urban	115	104	24	221
	Rural	0	64	157	243
	Total	0	168	181	464

Table 9: DHS and GHS-SMOD classification of urbanicity based on Cameroon's 2019 DHS clusters

		GHS-SMOD urbanicity classes- DHS 2019			
		Core urban	Semi urban	Rural	Total
DHS residence	Urban	150	121	24	295
	Rural	0	85	197	282
	Total	150	206	221	577

Zambia Case study

Box 1: DHS and GHS-SMOD classes

We investigated six (and their surroundings) of the 28 EAs categorised as urban by DHS but as rural by GHS-SMOD in Zambia. They are in Senanga (Figure 15, A-D) and Kaoma (Figure 15, E-H) towns with a population of 14,102 and 19,851 on the 2010 census, respectively. However, upon examining the GHS-SMOD imagery in Senanga, one EA was classified as urban, while the second was semi-urban, and the third one was in a sparsely populated rural area. This was supported by the Africapolis data (D) and partly by Google Maps (C). Our classification (B) categorized two clusters as peri-urban and one as rural, respectively, driven by using circular buffers to reduce the geo scrambling of clusters by the DHS program. Geo-scrambling may have played a role in Senanga, resulting in the misplacement of urban areas in a non-urban location. The distance between the cluster and the GHS-SMOD cells was less than the 2km threshold, which in fact supports this theory that the cluster was displaced from a more urban location (within 2 km buffer). Nevertheless, the DHS classification was still limited as the urban and semi-urban were not distinguished but conflated. Similar observations were made by Dorélien and colleagues [5]. Findings were the same in Kaoma town.

Figure 15: zoomed in towns in Zambia where there was a mismatch between DHS and GHS-SMOD classification.

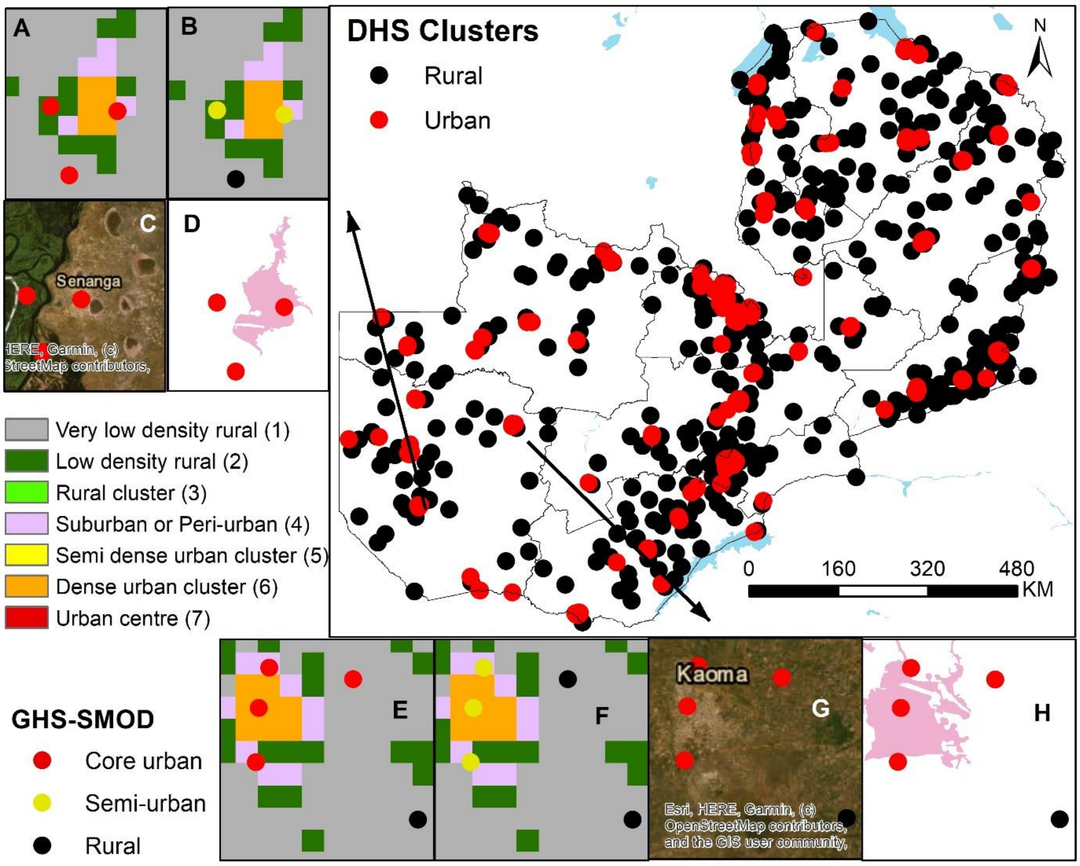

- *A and E: DHS cluster overlaid on the GHS-SMOD urbanization surface*
- *B and F: GHS-SMD derived classes overlaid on the GHS-SMOD urbanization surface*
- *C and G: DHS cluster overlaid on Google maps*
- *H and D: DHS cluster overlaid Africapolis urban areas*

Maternal health indicators

Table 10: Key maternal health indicators for Zambia's 2013/14 DHS used in the analysis

1	Percentage of women aged 15-49 who had a live birth in the five years preceding the survey who did not receive antenatal care (ANC) for the most recent birth.
2	Percentage of live births in the five years preceding the survey by place of delivery in a health facility (public or private), home or other places.
3	Among last live births not delivered in a health facility, percentage whose mothers cite specific reasons for not delivering in a facility (permission, money, distance and want to go alone)
4	Percentage of currently married women aged 15-49 using a current contraceptive method
5	Percentage of live births in the five years preceding the survey delivered by caesarean section

References

1 Fish TD, Janocha B, Dontamsetti T, *et al.* Predicting geospatial covariates:Proxies for mapping urban-related indicators.DHS Spatial Analysis Reports No. 19. Rockville, Maryland, USA: 2020.

2 Ghana Statistical Service. 2010 Population and Housing Census: Urbanisation in Ghana. 2014. https://statsghana.gov.gh/gssmain/fileUpload/pressrelease/Urbanisation%20in%20Ghana.pdf (accessed 17 Jun 2023).

3 Macharia PM, Mumo E, Okiro EA. Modelling geographical accessibility to urban centres in Kenya in 2019. *PLoS One* 2021;**16**. doi:10.1371/journal.pone.0251624

4 Iven M Sikanyiti. Expert Group Meeting on Statistical Methodology for Delineating Cities and Rural Areas. New York: 2019. https://unstats.un.org/unsd/demographic-social/meetings/2019/newyork-egm-statmeth/docs/s05-01-ZMB.pptx (accessed 17 Jun 2023).

5 Dorélien A, Balk D, Todd M. What is Urban? Comparing a satellite view with the demographic and health surveys. *Popul Dev Rev* 2013;**39**. doi:10.1111/j.1728-4457.2013.00610.x
